# Association of Air Pollution with Increased Incidence of Ventricular Tachyarrhythmias Recorded by Implanted Cardioverter Defibrillators

**DOI:** 10.1289/ehp.7767

**Published:** 2005-02-18

**Authors:** Douglas W. Dockery, Heike Luttmann-Gibson, David Q. Rich, Mark S. Link, Murray A. Mittleman, Diane R. Gold, Petros Koutrakis, Joel D. Schwartz, Richard L. Verrier

**Affiliations:** ^1^Department of Environmental Health, Harvard School of Public Health, Boston, Massachusetts, USA; ^2^Channing Laboratory, Brigham and Women’s Hospital and Harvard Medical School, Boston, Massachusetts, USA; ^3^New England Medical Center, Tufts University, Boston, Massachusetts, USA; ^4^Department of Epidemiology, Harvard School of Public Health, Boston, Massachusetts, USA; ^5^Beth Israel Deaconess Medical Center and Harvard Medical School, Boston, Massachusetts, USA

**Keywords:** air pollution, arrhythmias, epidemiology, fibrillation, heart arrest

## Abstract

Epidemiologic studies have demonstrated a consistent link between sudden cardiac deaths and particulate air pollution. We used implanted cardioverter defibrillator (ICD) records of ventricular tachyarrhythmias to assess the role of air pollution as a trigger of these potentially life-threatening events. The study cohort consisted of 203 cardiac patients with ICD devices in the Boston metropolitan area who were followed for an average of 3.1 years between 1995 and 2002. Fine particle mass and gaseous air pollution plus temperature and relative humidity were measured on almost all days, and black carbon, sulfate, and particle number on a subset of days. Date, time, and intracardiac electrograms of ICD-detected arrhythmias were downloaded at the patients’ regular follow-up visits (about every 3 months). Ventricular tachyarrhythmias were identified by electrophysiologist review. Risk of ventricular arrhythmias associated with air pollution was estimated with logistic regression, adjusting for season, temperature, relative humidity, day of the week, patient, and a recent prior arrhythmia. We found increased risks of ventricular arrhythmias associated with 2-day mean exposure for all air pollutants considered, although these associations were not statistically significant. We found statistically significant associations between air pollution and ventricular arrhythmias for episodes within 3 days of a previous arrhythmia. The associations of ventricular tachyarrhythmias with fine particle mass, carbon monoxide, nitrogen dioxide, and black carbon suggest a link with motor vehicle pollutants. The associations with sulfate suggest a link with stationary fossil fuel combustion sources.

A large number of epidemiologic studies have found an association between short-term episodes of increased particulate air pollution and cardiovascular morbidity and mortality (Brook et al. 2004). Respirable particulate matter has been specifically implicated in the triggering of myocardial infarction (D’Ippoliti et al. 2003; Peters et al. 2001), arrhythmias (Peters et al. 2000), decompensation of heart failure patients (Morris and Naumova 1998; Schwartz and Morris 1995; Wellenius et al., in press), and the exacerbation of myocardial ischemia (Pekkanen et al. 2002; Wellenius et al. 2003). Particulate-related changes in autonomic nervous system activity, as assessed by heart rate variability, have been observed in both experimental animal studies (Godleski et al. 2000) and human panel studies (Creason et al. 2001; Gold et al. 2000; Liao et al. 1999, 2004; Pope et al. 1999), suggesting sympathetic activation or vagal suppression after particulate air pollution exposure. Such changes in autonomic tone may increase the risk of ventricular arrhythmias in vulnerable patients (Huikuri et al. 2001). Ventricular tachyarrhythmias, primarily ventricular tachycardia and ventricular fibrillation, are common precursors to sudden cardiac death (Bayes de Luna et al. 1989; Myerburg et al. 1992).

Implanted cardioverter defibrillators (ICDs) passively monitor for ventricular tachyarrhythmias that, if not terminated, could be life threatening. On detecting such an arrhythmia, the ICD can apply cardiac pacing or cardioverter shock to restore normal rhythms. The ICD also records the date and time of arrhythmias plus intracardiac electrograms immediately before and during these events. In a pilot study of 100 Boston area ICD patients with follow-up for up to 3 years, we found increased risk of an ICD therapeutic discharge on days after elevated air pollution concentrations (Peters et al. 2000). In this pilot study, we did not collect data on patient characteristics or medication. However, we did find stronger air pollution associations among patients with frequent ICD discharges.

This study was designed to confirm the pilot study observations. In a larger sample of ICD patients in Boston with longer follow-up, we identified ventricular tachyarrhythmias by review of ICD-recorded electrograms. We assessed the association between community air pollution and ventricular tachyarrhythmias using time-series methods. We also evaluated modification of the air pollution association by patient medical conditions, antiarrhythmic medications, and recent arrhythmias.

## Materials and Methods

### Arrhythmias.

We examined the effects of air pollution on incidence of tachyarrhythmias in ICD patients clinically followed between July 1995 and July 2002 at the Tufts New England Medical Center (Dockery et al., in press). The source population consisted of patients implanted with third-generation Guidant ICDs (Cardiac Pacemakers, Inc., St. Paul, MN) at the New England Medical Center Cardiac Electrophysiology and Pacemaker Laboratory between June 1995 and 31 December 1999. All patients met the American College of Cardiology and the American Heart Association guidelines for ICD implantation (Gregoratos et al. 1998). We excluded patients residing in ZIP codes > 40 km (25 miles) from the air monitoring site at the Harvard School of Public Health. Patient characteristics before implant (including age, sex, race and ethnicity, residential ZIP code, implant date, device model, diagnoses at implant, and physiologic measurements before implant) were abstracted from patient records. Prescribed medications were abstracted from clinical records at each follow-up visit.

Date, time, and intracardiac electrograms of all detected arrhythmias were downloaded from the ICD records collected at the patients’ regular clinical follow-up visits (on average, every 89 days). Patients contributed person-time to the follow-up between ICD implantation and their last clinical follow-up visit at the New England Medical Center before July 2002. We excluded the first 14 days after implantation, periods when the patient was a hospital inpatient, and periods between clinical visits when the patient was not followed up at the New England Medical Center. Subjects who died or who were lost to follow-up were censored at their last clinical follow-up.

The intracardiac electrograms for each ICD-detected arrhythmia were reviewed by an electrophysiologist (M.S.L.) blinded to air pollution levels. Ventricular tachyarrhythmias were identified based on atrial-ventricular dysynchrony, onset interval, stability, morphology of the tachycardia, and response to therapy. We excluded sinus tachycardias, arrhythmias originating outside the ventricle (e.g., atrial tachycardia, atrial fibrillation, atrial flutter, sinus tachycardia), and noise or over-sensing events. An episode day was defined as one or more ventricular arrhythmic events on a given calendar day.

Data collection and preliminary analyses have been described previously (Dockery et al., in press). The Harvard School of Public Health Human Studies Committee and the New England Medical Center Institutional Review Board approved this retrospective record review.

### Air pollution.

Ambient concentrations of gaseous air pollutants were measured by the Massachusetts Department of Environmental Protection between 1995 and 2002 at six sites for ozone, nitrogen dioxide, and/or sulfur dioxide and four sites for carbon monoxide in the Boston metropolitan area. We calculated the average air pollution concentration across the reporting air pollution monitoring stations for each hour accounting for differences in the annual mean and the standardized deviations of each monitor (Schwartz 2000). The daily mean was then calculated from the 24-hr specific average concentrations across the monitors.

Fine particulate (< 2.5 μm aerodynamic diameter) matter (PM_2.5_) concentrations were measured (model 1400A tapered element oscillating microbalance; Rupprecht and Patashnick, East Greenbush, NY) at an ambient monitoring site in South Boston between 15 January 1995 and 19 January 1998 and at the Harvard School of Public Health starting on 17 March 1999. Particulate black carbon (BC) was measured (aethalometer model 8021; McGee Scientific, Berkeley, CA) at the South Boston site through 29 March 1997 and at the Harvard School of Public Health site starting on 15 October 1999. Daily particulate sulfate (SO_4_) was measured by ion chromatography (model 120; Dionex, Sunnyvale, CA) starting on 25 September 1999, and particle number (PN) by condensation particle counter (TSI Inc., Shoreview, MN) starting on 13 October 1999. We did not consider PM_10_ (particulate matter with a diameter < 10 μm), which was measured on a 1-in-6–day schedule.

The hourly surface observations from the National Weather Service at Logan Airport in East Boston were extracted from climatic records (Earth-Info, Inc., Boulder, CO). Daily minimum temperature and mean relative humidity were calculated from the hourly observations.

### Statistical analyses.

Following the analytic methods used in the pilot study (Peters et al. 2000), we assessed the association of arrhythmias with air pollution using time-series methods. We merged the patient-specific record of days on study and ICD-detected ventricular arrhythmias with the daily mean air pollution and weather measurements. The association of arrhythmic episode-days with air pollution was analyzed by logistic regression using generalized estimating equations (Diggle 1988; Zeger et al. 1988) with random effects for patients, a linear trend, sine and cosine terms with periods of one, one-half, one-third, and one-quarter year, quadratic functions of minimum temperature and mean humidity, indicators for day of the week, and an indicator for a previous arrhythmia within 3 days.

We considered mean air pollution concentrations on the same day and lags of 1, 2, and 3 days. The lag structure of the data was estimated by evaluating each lag day (0 to 3) separately and jointly in an unconstrained distributed lag model (Pope and Schwartz 1996). We have found consistently elevated (although not statistically significant) risk estimates associated with air pollution concentrations on the day of (lag 0) and the day before (lag 1) the arrhythmia (Dockery et al., in press). Therefore, in this article we report only the effects of 2-day running mean air pollution concentrations.

We explored potential modification of the air pollution associations in multivariate logistic regression including interactions between air pollution and indicators of patient characteristics. Patients were stratified by reported ejection fraction before implantation (≤35% vs. > 35%), prior myocardial infarction, and the diagnosis of coronary artery disease before implantation (not sufficient numbers of patients for specific analyses of other cardiac diagnoses). We assessed modification of the air pollution associations by usual prescribed medications (reported at more than half of clinical follow-ups) grouped as beta-blockers, digoxin, and other antiarrhythmics (amiodarone, sotalol, mexilitine, and quinidine). The strongest predictor of a ventricular arrhythmia was an arrhythmia in the previous 3 days. Therefore, in addition to controlling for prior arrhythmias, we assessed the modification of the air pollution association by a prior ventricular arrhythmia.

We present odds ratios (ORs) and 95% confidence intervals (CIs) based on an interquartile range (25th percentile–75th percentile) increase in each air pollution concentration. *p*-Values are reported for the effects of air pollution and for the interactions of air pollution with posited effect modifiers. We characterize *p*-values < 0.05 as statistically significant, and *p*-values < 0.10 as marginally significant. For air pollutants and subgroups of events with statistically significant associations, we examined the risk of arrhythmias versus quintiles of air pollution concentration.

## Results

### Patient population.

A total of 307 patients had Guidant ICDs implanted at the New England Medical Center between June 1995 and the end of 1999. There were 203 patients followed up with residential ZIP codes within 40 km (25 miles) of the ambient air pollution monitoring site at the Harvard School of Public Health. These ICD patients had a total of 635 person-years (pyr) of follow-up or an average of 3.1 years per subject.

There were 933 ICD-detected tachyarrhythmias (separated by at least 60 min), of which 798 (86%) were ventricular (63 ventricular fibrillation, 25 nonsustained ventricular fibrillation, 622 ventricular tachycardia, and 88 nonsustained ventricular tachycardia). We restricted analysis to the 670 ventricular episode days (one or more ventricular arrhythmias on a calendar day), average of 1.06 episode days/pyr, among 84 (41%) patients.

Patients were predominantly men (75%) with an average age at implantation of 64 years (range, 19–90 years). The rate of ventricular episode days per person-year was higher among men (1.22/pyr) compared with women (0.62/pyr), and increased with age at implantation. Eighty-three percent of the patients were reported to be white, 3% African American, 5% Hispanic, 3% Asian, and 7% of undetermined or unknown race/ethnicity.

Among the patients reported to have had a myocardial infarction before ICD implantation, the rate of ventricular arrhythmias (1.73/pyr) was almost three times the rate among the patients without a prior myocardial infarction (0.61/pyr). The patients with low ejection fraction (≤35%) before implantation had a rate of ventricular episodes (1.48/pyr) approximately three times larger than that of patients with ejection fraction > 35% (0.45/pyr).

The most common preimplantation diagnosis was coronary artery disease (70%) followed by cardiomyopathy (36%). Nine patients (4%) were classified as having primary electrical disease, and four of these had ventricular arrhythmic events. Four patients (2%) had long QT syndrome (a congenital disorder characterized by prolongation of the QT interval on the electrocardiogram), but only one of these had an event during follow-up. Patients with coronary artery disease had the highest rates of detected ventricular arrhythmias (1.30/pyr) compared with those with other diagnoses (0.50/pyr).

Eighty-nine percent of these patients were prescribed antiarrhythmic medications. The rates of ventricular arrhythmic episode days was higher among those prescribed digoxin (1.68/pyr) or other antiarrhythmics (1.45/pyr) than among those prescribed beta-blockers (0.92/pyr) or no regular antiarrhythmic medication (0.88/pyr).

Approximately one-quarter (164) of the 670 ventricular arrhythmias followed a previous ventricular arrhythmia within 3 days. We found that having a prior arrhythmia (within 3 days) was a very strong predictor for a subsequent arrhythmia (OR = 7.2; 95% CI, 5.9–8.9).

### Air pollution.

PM_2.5_ was measured on 2,005 (79%) of the follow-up days and BC on 1,533 (60%) days (Table 1). Particulate SO_4_ measurements were limited to 908 (36%) days, and PN to 772 (30%) days. Daily PM_2.5_ was strongly correlated with SO_4_ (Pearson correlation *r* = 0.74) and BC (*r* = 0.67), but only weakly correlated (*r* = –0.13) with PN.

The gaseous pollutants were measured on essentially all follow-up days (Table 1). Daily CO and NO_2_, both indicators of motor vehicle emissions, were highly correlated with each other (*r* = 0.61), positively correlated (*r* > 0.4) with BC, PM_2.5_, and SO_2_, but negatively correlated with O_3_.

### Air pollution association.

We found positive associations between ventricular arrhythmic episode days and mean air pollution on the same and previous days, but none of these associations approached statistical significance (Table 2).

We did not find consistent increased susceptibility to the effects of air pollution on risk of ventricular arrhythmias based on patient characteristics. We found marginally significant (*p* < 0.10) interaction of the associations with CO with ejection fraction (stronger with low ejection fraction), preimplantation diagnosis of coronary artery disease (weaker with coronary artery disease), and prior myocardial infarction (weaker with prior myocardial infarction), and of the associations with NO_2_ with prior myocardial infarction (stronger with prior myocardial infarction). No other interactions approached statistical significance. We saw no evidence that any of the prescribed drugs modified the associations of ventricular arrhythmias with air pollution.

**Figure 1:**
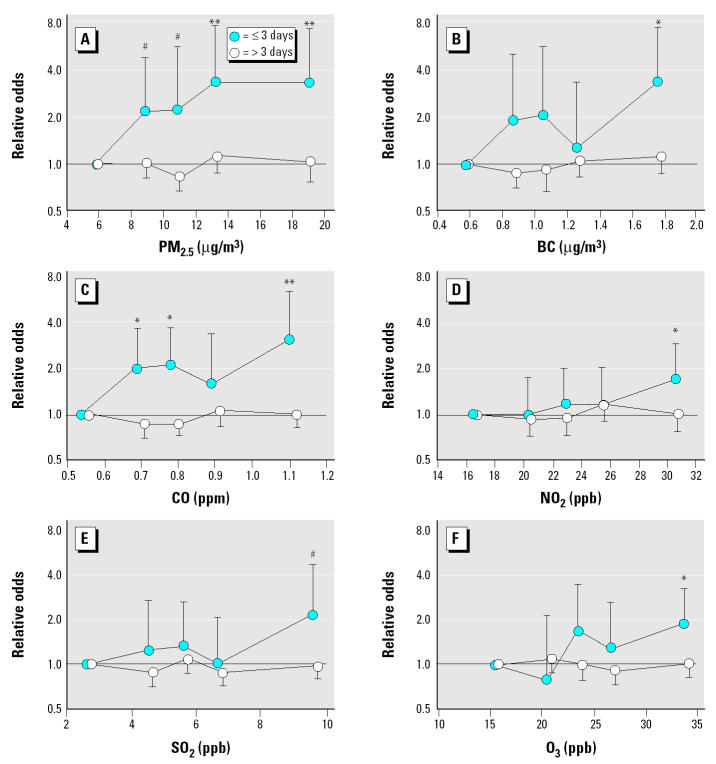
Relative odds and 95% CIs of ventricular arrhythmias versus quintiles of air pollution, ≤3 days of and > 3 days after, a previous arrhythmia: (*A*) PM_2.5_, (*B*) BC, (*C*) CO, (*D*) NO_2_, (*E*) SO_2_, (*F*) O_3_.
^#^*p* < 0.10;
*p < 0.05;
**p < 0.01.

The interaction of a prior ventricular arrhythmia with air pollution was statistically significant for PM_2.5_, BC, NO_2_, SO_2_, and CO and marginally significant for SO_4_ (Table 3). For ventricular arrhythmias within 3 days of a prior event (Table 3), we found statistically significant positive associations with PM_2.5_, BC, NO_2_, CO, and SO_2_, marginally significant associations with SO_4_, but no associations with O_3_ or PN. For ventricular arrhythmias more than 3 days after a previous ventricular arrhythmia, we found no associations with any air pollutants (Table 3). We assessed the risk of ventricular arrhythmias stratified by a prior ventricular tachyarrhythmia versus quintiles of air pollution (Figure 1). We found generally increasing risk with increasing quintiles of PM_2.5_, BC, and CO and weaker suggestions of an exposure response with NO_2_, SO_2_, and O_3_.

## Discussion

In this study of 203 New England Medical Center ICD patients living in the Boston metropolitan area with up to 7 years of follow-up, we found the risk of any ICD-detected ventricular tachyarrhythmia was positively but not significantly associated with increased exposure to air pollution on the days before the arrhythmia (Table 2). We found statistically significant associations of air pollution with increased risk of ventricular arrhythmias among patients with an arrhythmia within the previous 3 days. These findings suggest that air pollution may provoke ventricular tachyarrhythmias only in the presence of acutely predisposing conditions that increase ventricular electrical instability. We did not find consistent indications that the air pollution associations with ventricular arrhythmias were modified by indicators of chronically impaired cardiac function, including a prior myocardial infarction, a diagnosis of coronary artery disease, or an ejection fraction < 35%, or by prescribed antiarrhythmic medications.

These results are broadly consistent with those of previously published studies of air pollution associations with tachyarrhythmias leading to ICD therapeutic discharge. In this study, we found significantly increased risk of ventricular arrhythmias with PM_2.5_, BC, CO, NO_2_, and SO_2_ among patients with a recent rior ventricular arrhythmia. In the pilot study (Peters et al. 2000), ICD patients in Boston with frequent (> 10) discharges during follow-up had an exposure related increase in ICD discharge associated with PM_2.5_, BC, CO, and NO_2_.

A recent study assessed the association of air pollution in Vancouver, British Columbia, Canada, with ICD discharges among 50 patients with an average of 2.2 years of follow-up (Rich et al. 2004; Vedal et al. 2004). In crude analyses, the rate of ICD discharge increased with quartiles of NO_2_ and CO concentration on the same day (Vedal et al. 2004). However, there were no statistically significant positive associations with ICD discharge with NO_2_ or CO after adjusting for temporal patterns and numerous weather parameters. The lack of significant associations may be caused by overcontrol of some variables, as these investigators suggest.

Both of these previously reported studies (Peters et al. 2000; Vedal et al. 2004) focused on ICD therapeutic discharge without characterization or validation of the underlying arrhythmia. Of the almost 2,000 arrhythmias identified and recorded by the ICD devices in this study, 8% were classified as oversensing, 4% were sinus tachycardias, 18% were supra-ventricular arrhythmias, and 70% were ventricular arrhythmias. Thus, 30% of the ICD-detected arrhythmias were not the potentially life-threatening ventricular tachyarrhythmias defined as the primary outcome for this study.

An important question in these analyses is the appropriate exposure averaging time and the lag between exposure and cardiac arrhythmia. In the pilot study, we found associations with air pollutants 2 days before the arrhythmias and with the 5-day mean air pollution (Peters et al. 2000). In this study, ventricular arrhythmias were positively associated with ambient air pollution on the same and the previous calendar days. Temporality would require that air pollution exposure precede the arrhythmia. This temporal association is clearly true for associations with air pollution on the previous day, but mean air pollution on the same calendar day would include hours after as well as before the detected arrhythmia. Using the pollutant concentrations from the specific 24 hr preceding the arrhythmia would likely provide a better estimate of each subject’s exposure and allow investigation of exposures in the hours before the arrhythmia.

For these patients living in eastern Massachusetts, air pollution exposure was estimated based on a single or a small number of monitors in the Boston metropolitan area. This would lead to misclassification of air pollution exposure, but this misclassification would be independent of the risk for ventricular arrhythmias. Such nondifferential misclassification of exposure produces an attenuated estimate of associations (and larger CIs) in epidemiologic studies assuming linear associations. If these observations are true, then studies with improved estimation of subject specific air pollution exposures would be expected to find stronger, more statistically significant associations.

We found associations with CO, NO_2_, BC, and PM_2.5_. These four pollutants had high day-to-day correlations with each other and were strongly correlated with SO_2_. It would not be possible to differentiate the independent effects of these pollutants. Nevertheless, the associations with these specific pollutants are consistent with an effect from air pollution from motor vehicle sources.

Animal studies in Boston have suggested that changes in indicators of cardiac function are specifically associated with motor vehicle pollution (Clarke et al. 2000). Analysis of daily mortality in Boston and five other cities suggested that motor vehicle pollution was more strongly related to cardiovascular mortality than to respiratory mortality (Laden et al. 2000). Cardiovascular emergency department visits in Atlanta, Georgia, were significantly associated with these same markers of motor vehicle air pollution—NO_2_, CO, PM_2.5_, BC, and fine particle organic carbon (Metzger et al. 2004). For Atlanta emergency department visits for dysrhythmias, positive associations were found for these same motor vehicle pollutants, although these associations were not statistically significant because of the smaller number of events.

We cannot exclude the possible role of sulfur oxides, which are generally considered to be indicators of air pollution from power plants and other stationary fossil fuel combustion sources. In this analysis, we found associations of ventricular tachyarrhythmias in subjects with a recent event associated with SO_2_ (*p* = 0.013) and with SO_4_ (*p* = 0.06). The positive, marginally significant associations with SO_4_ are notable because SO_4_ data were available only on a limited number of days (37%) compared with SO_2_ and the other gaseous pollutants. Particulate SO_4_ concentrations in Boston largely reflect secondary particles formed during long-range transport. Gaseous SO_2_ concentrations reflect local sulfur emissions and were most highly correlated with motor vehicle pollutants.

A major advantage of the ICD data is the passive monitoring of cardiac tachyarrhythmias. Nevertheless, ICD-detected ventricular arrhythmias were rare events in this follow-up, and the small number of subjects with multiple ICD-detected arrhythmias is a limitation. These patients clearly represent a highly selected cohort of special interest, because their previous history of cardiovascular disease might make them particularly sensitive to the effects of air pollution episodes. The observed associations of ventricular tachyarrhythmias with particulate air pollution in these subjects are large compared with previous studies. In a mortality time-series analysis in Boston and five other cities (Schwartz et al. 1996), each increase of 10 μg/m3 in the 2-day mean PM_2.5_ was associated with a 2% increase in the risk of cardiovascular mortality. For Boston ICD patients (Table 2), the observed associations imply an 11% (95% CI, –9 to 35%) increased risk of potentially fatal ventricular arrhythmias when scaled to the same 10 μg/m^3^ in the 2-day mean PM_2.5_ concentrations. Thus, the ICD patients had a risk of potentially life-threatening ventricular tachyarrhythmias associated with fine particles that was more than five times the risk of cardiovascular death in the general population. Among those at the highest risk—those with a recent prior ventricular arrhythmia—the increased risk of a new ventricular tachyarrhythmia was 97% (95% CI, 46–165%) associated with each 10-μg/m^3^ increase in PM_2.5_.

## Conclusions

We found that ventricular tachyarrhythmias among patients with ICDs increased with air pollution on the same and previous days, but these associations did not reach statistical significance. However, among patients with a recent tachyarrhythmia, the increased risk of a follow-up ventricular tachyarrhythmia associated with air pollution was large and statistically significant. These observations suggest that air pollution may act in combination with a cardiac electrical instability to increase the risk for ventricular tachyarrhythmias. Among such acutely vulnerable ICD patients, there was an exposure response with PM_2.5_, BC, NO_2_, CO, and SO_2_, which we interpret as indicators of mobile source pollution, and also evidence of an association with SO_4_, which we interpret as an indicator of power plant and other stationary fossil fuel combustion sources.

ICDs have proven to be highly effective in reducing the risk of death in patients with high risk of cardiac arrhythmias. The passive monitoring of arrhythmias by these devices has provided a rich resource for understanding the role of air pollution episodes as potential triggers of these events.

## Correction

Some counts of observations (Tables 1 and 2) and the interquartile range of SO_4_ (Tables 1, 2, and 3) were for 1-day rather than 2-day mean in the original manuscript published online. They have been corrected here.

## Figures and Tables

**Table 1:** Distribution of the 2-day mean air pollutants averaged across multiple sites in Boston, and weather data: 11 July 1995 to 11 July 2002.

		Percentile			
Air pollutant	No.	25th	50th	75th	95th
PM_2.5_ (μg/m^3^)	2,005	7.5	10.3	14.4	23.3
BC (μg/m^3^)	1,533	0.66	0.98	1.39	2.25
SO_4_ (μg/m^3^)	908	1.76	2.55	3.80	7.18
PN (10^3^/cm^3^)	772	20.6	29.3	39.8	50.7
NO_2_ (ppb)	2,556	18.9	22.7	26.6	33.6
CO (ppm)	2,558	0.53	0.80	1.02	1.37
SO_2_ (ppb)	2,558	3.3	4.9	7.4	12.8
O_3_ (ppb)	2,548	15.7	22.9	31.1	42.1
Minimum temperature (°C)	2,553	0.6	7.2	14.4	20.6
Relative humidity (%)	2,549	56.7	69.0	81.5	94.3

**Table 2:** Estimated ORs (95% CIs) for an interquartile range increase in 2-day mean air pollution.

	No. of days	Interquartile range increase	OR (95% CI)	*p*-Value
PM_2.5_	2,005	6.9 μg/m^3^	1.08 (0.96–1.22)	0.21
BC	1,533	0.74 μg/m^3^	1.11 (0.95–1.28)	0.18
SO_4_	908	2.04 μg/m^3^	1.05 (0.92–1.20)	0.48
PN	772	19,120/cm^3^	1.14 (0.87–1.50)	0.35
NO_2_	2,556	7.7 ppb	1.07 (0.97–1.18)	0.19
CO	2,558	0.48 ppm	1.14 (0.95–1.29)	0.28
SO_2_	2,558	4.0 ppb	1.04 (0.94–1.14)	0.28
O_3_	2,548	15 ppb	1.09 (0.93–1.29)	0.28

**Table 3:** Association of interquartile range increase in 2-day mean air pollution with ventricular arrhythmias stratified by a recent arrhythmia (within 3 days).

Air pollutant (IQR increase)	> 3 Days	< 3 Days	*p*-Value for interaction
PM_2.5_ (6.9 μg/m^3^)	0.98 (0.86–1.12) *p* = 0.73	1.60 (1.30–1.96) *p* < 0.001	< 0.001
BC (0.74 μg/m^3^)	1.02 (0.83–1.24) *p* = 0.86	1.74 (1.28–2.37) *p* < 0.001	0.003
SO_4_ (2.04 μg/m^3^)	1.03 (0.87–1.22) *p* = 0.73	1.19 (0.99–1.43) *p* = 0.060	0.066
PN (19,120/cm^3^)	1.17 (0.82–1.66) *p* = 0.38	1.11 (0.71–1.75) *p* = 0.65	0.86
NO_2_ (7.7 ppb)	1.02 (0.90–1.16) *p* = 0.78	1.34 (1.05–1.71) *p* = 0.018	0.050
CO (0.48 ppm)	1.04 (0.83–1.29) *p* = 0.75	1.65 (1.17–2.33) *p* = 0.005	0.016
SO_2_ (4 ppb)	0.98 (0.87–1.11) *p* = 0.78	1.30 (1.06–1.61) *p* = 0.013	0.006
O_3_ (15 ppb)	1.14 (0.92–1.40) *p* = 0.24	1.01 (0.76–1.35) *p* = 0.94	0.44
